# The association of safety-net program participation with government perceptions, welfare stigma, and discrimination

**DOI:** 10.1093/haschl/qxad084

**Published:** 2023-12-21

**Authors:** Richard Pulvera, Kaitlyn Jackson, Wendi Gosliner, Rita Hamad, Lia C H Fernald

**Affiliations:** University of California Agriculture and Natural Resources, Nutrition Policy Institute,Oakland, CA 94607, United States; T.H. Chan School of Public Health, Harvard University,Boston, MA 02115, United States; University of California Agriculture and Natural Resources, Nutrition Policy Institute,Oakland, CA 94607, United States; T.H. Chan School of Public Health, Harvard University,Boston, MA 02115, United States; School of Public Health, University of California Berkeley,Berkeley, CA 94704, United States

**Keywords:** SNAP, EITC, safety-net programs, attitudes, stigma, discrimination

## Abstract

Safety-net programs in the United States offered critical support to counter food insecurity and poverty during the first year of the COVID-19 pandemic. The Supplemental Nutrition Assistance Program (SNAP) and the Earned Income Tax Credit (EITC) are both means-tested programs with significant benefits. Take-up of SNAP and EITC is lower in California than nationwide and reasons for this difference are unclear. We examined associations of participation in SNAP and receipt of the EITC and perceptions of the US government, 2 types of welfare stigma (program stigma and social stigma), and perceived discrimination. We interviewed a sample of 497 caregivers of young children from families with low income in California during the COVID-19 pandemic (August 2020-May 2021). We found that participation in SNAP (odds ratio [OR] = 1.24 [1.05, 1.47]) and receiving the EITC (OR = 1.39 [1.05, 1.84]) were both associated with greater reported perceptions of social stigma, but not with perceptions of government, program stigma, or discrimination. Among food-insecure respondents, we found that participation in SNAP was additionally associated with program stigma and discrimination. These findings suggest that perceived social stigma may be a reason that people with low income may not participate in programs for which they are eligible.

## Introduction

Safety-net programs in the United States provide critical support to families facing economic hardship and food insecurity. The number of families experiencing hardship has increased in recent years: in 2020, 11.4% of the US population (37.2 million people) lived below the poverty level, up from 10.5% in 2019.^1^ The poverty rate among children was even greater at 16.1% in 2020, up from 14.4% in 2019.^1^ Food insecurity also worsened through the early months of the COVID-19 pandemic.^2-4^ In 2020, 14.8% of US households with children were food insecure, meaning that, at some time during the year, the household had difficulty providing adequate, nutritious foods to all members,^5^ which was up from 13.6% in 2019.^6^ Food insecurity is associated with adverse health outcomes, including increased diabetes, hypertension, and mental health problems among adults.^7-13^

Specific safety-net programs offer targeted assistance to households to reduce food insecurity. For example, the Supplemental Nutrition Assistance Program (SNAP; formerly known as “food stamps”) offers funds that households can use to buy groceries.^14^ SNAP participants receive monthly benefits through an electronic benefit transfer (EBT) card that can be used at authorized retailers.^14^ SNAP is the largest domestic food and nutrition assistance program for Americans with low incomes and is especially responsive during economic downturns when more people become eligible.^15^ Meanwhile, the Earned Income Tax Credit (EITC) aims to alleviate financial hardship by offering assistance to low- and moderate-income earners through an annual lump-sum tax refund distributed to those who file taxes and claim the benefit.^16^ The EITC is one of the largest US anti-poverty programs, distributing more than $60 billion to approximately 25 million families each year.^17^ Unlike SNAP, this program is less responsive to shocks because tax filing and disbursement occurs on a fixed annual schedule. Eligibility criteria for these and other programs vary, but typically include a combination of earned income, household composition, and citizenship or residency status, among other criteria. In response to the COVID-19 pandemic, these and other programs were temporarily expanded to respond to emergent needs. In March 2020, Congress temporarily increased SNAP benefits to the allowable household maximum for all participants and approved online SNAP purchases at specific retailers.^18^ Changes to the EITC included offering applicants the option of using 2019 or 2020 earned income for 2020 tax filing, enabling more filers to become eligible.^18^

Both SNAP and the EITC have well-documented health benefits.^19-23^ However, take-up and demographics vary by program and setting. In 2019, California ranked 46th in the nation for SNAP take-up, with 70% of all SNAP-eligible people participating compared to 82% nationally.^24^ Among participating households, 42% of SNAP households had children in California compared to 40% nationally in 2019.^25^ California's take-up of the EITC is similar, with the state ranking among the lowest. In 2019, 74.5% of EITC-eligible filers in California received the benefit compared to 79.3% of filers nationally.^26^ Take-up of these programs may be associated with a number of social experiences and beliefs, which we discuss below, including potential recipients' (1) perceptions about government, (2) perceptions of welfare stigma, and (3) experiences with discrimination.

Personal moral values, party affiliation, and political ideology, among other factors, inform an individual's perceptions of the roles of government, which may then influence participation in safety-net programs. According to a nationally representative survey conducted in 2020 by Pew Research Center, only 36% of Americans overall have a positive view about the government regarding poverty alleviation, with a sharp partisan divide between Republicans (59%) and Democrats (18%).^27^ In contrast, participation in safety-net programs is a much more bipartisan experience. A different Pew Research Center survey from 2012 found modest differences in take-up by party affiliation; 60% of Democrats, 52% of Republicans, and 53% of independents have ever benefited from any of the major safety-net programs.^28^ Take-up is also similar by political ideology, with conservatives (57%), liberals (53%), and political moderates (53%) all having benefited from any of the major safety-net programs at similar percentages.^28^ This suggests that neither party affiliation nor political ideology are key drivers of safety-net program participation at the national level. A US Department of Agriculture (USDA) Economic Research Service report cites other reasons for not participating in SNAP among eligible nonparticipants, such as a desire for personal independence (91%), a desire for privacy (25%), and previous bad experiences with government programs (24%).^29^

Stigma related to welfare participation may also impact take-up. From the same USDA report, 45% of eligible nonparticipants expressed that stigma was another deterrent from participating in SNAP.^29^ We discuss 2 forms of stigma: (1) program stigma and (2) social stigma. Program stigma originates from the process of applying for or participating in safety-net programs. Burdensome application processes for safety-net programs can impart psychological costs on potential beneficiaries. For example, many applicants cite reluctance to answer intrusive personal questions as well as poor treatment or indifference from case workers as reasons for not participating.^30^ SNAP-eligible nonparticipants also cite costs of applying (eg, paperwork, time away from work or dependent care, transportation; 64%), work requirements (17%), and general confusion about the application process (12%).^29^ US safety-net program rules require a high degree of learning and strict compliance.^31,32^ Another type of stigma, social stigma, originates from the anticipation of judgment from others. A prior study found that 45% of SNAP-eligible nonparticipants expressed that they did not want to be seen shopping with SNAP, did not want others to know that they needed financial assistance, and did not want to go to the welfare office.^29,30^ In the United States, stigma is greatest when individuals are perceived to be responsible for their own economic hardship. Yet, people with limited incomes are often financially vulnerable due to structural barriers rather than individual decision making. Social stigma perpetuates the narrative that safety-net program participants are lazy, undeserving, and lacking ambition or morality.^33-35^ Just the anticipation of this characterization can negatively impact potential beneficiaries' confidence and willingness to seek assistance.

Experiences of discrimination may also impact take-up. Structural racism produces and perpetuates racial discrimination through the institutions with which individuals interact, including the welfare system. Structural racism is a fundamental cause of food insecurity, not only through disparities in income and generational wealth but also directly and independent of socioeconomic resources.^36,37^ In some cases, the implementation of safety-net programs is driven by a long history of racially motivated narratives that portray people of color as the face of poverty, although the largest group of beneficiaries identify as non-Hispanic White. Among all SNAP participants in 2019, the 3 largest race and ethnicity groups were non-Hispanic White (36.5%), non-Hispanic African American (25.8%), and Hispanic of any race (16.0%).^38^ Black women and families, in particular, have faced persistent discrimination through narratives centered on reliance on government assistance and abuse of benefits.^36^ Media coverage and popular literature perpetuate these narratives by disproportionately featuring Black Americans in stories about poverty.^39^ These false narratives continue to inform program rules, often to justify proposed funding cuts and benefits reductions.^40^ False narratives, racist imagery, and restrictive program rules—in conjunction with pervasive stigma—may leave eligible families feeling discouraged and targeted in pursuing assistance.

Our study aims to examine these related factors by assessing the associations of perceptions of government, welfare stigma, and discrimination with participation in SNAP and receipt of the EITC. To achieve this goal, we used data from a cross-sectional study that surveyed caregivers of young children from families with low income in California during the early part of the COVID-19 pandemic. We examined SNAP and EITC because of their importance in the safety-net system, and because of how differently they operate, which, we hypothesized, may lead to different correlates with program take-up.

## Data and methods

### Study design and sample

Our study aims to examine the associations of participation in SNAP and receipt of the EITC with perceptions of government, welfare stigma, and discrimination. The Assessing California Communities' Experiences with Safety Net Supports (ACCESS) study was a cross-sectional study, surveying caregivers of young children in California between August 2020 and May 2021 (*n* = 497). The ACCESS study sought to recruit survey respondents eligible for the EITC, many of whom would also be eligible for nutrition-assistance programs such as SNAP. Inclusion criteria were having at least 1 dependent age 0–8 years, earnings within eligibility limits for the EITC based on household size, and immigration status consistent with eligibility rules. Eligibility criteria, recruitment methods, and respondent screening for the ACCESS study have been described in detail previously.^41^ Briefly, we recruited our sample in partnership with community-based organizations including safety-net programs such as the Special Supplemental Nutrition Program for Women, Infants, and Children (WIC), social services agencies, tax preparation services, and other local direct service organizations. These community partners conducted outreach via recruitment e-mails, social media, newsletters, and text messages to their clients. Respondents were also asked to share study information with friends and family members, incorporating a snowball sampling methodology. Interviews were conducted using video-conferencing software or by telephone. They were 1.5 hours in length, on average, and consisted of a variety of topics, including sociodemographic characteristics, household composition, participation in and perceptions of safety-net programs, household income and EITC data collected from tax returns (for those who had filed taxes), and experiences with stigma and discrimination.

For SNAP analyses, the sample was restricted to those eligible for SNAP. For EITC analyses, the sample was restricted to those eligible for the EITC. We imputed respondents' eligibility for SNAP at the time of interview using self-reported data on household composition, including dependents, immigration status of the survey respondent, household income (including income from a spouse, if applicable), and concurrent participation in other safety-net programs that make one adjunctively eligible for SNAP. We verified respondents' eligibility and receipt of the EITC for tax year 2019 by asking them to share several key details that we confirmed from their tax returns, including tax filing status, number of dependents claimed, household earnings, and EITC refund amount (if received). Application of these additional inclusion criteria resulted in analytic subsamples of differing sizes: 489 SNAP-eligible study respondents and 326 EITC-eligible study respondents.

### Variables

We asked survey respondents about their perceptions of government, welfare stigma, and experiences of discrimination. Multiple items were used to measure each of these constructs (Table S1). We constructed our variables for government perceptions using items from the “Values About Government and Social Safety Net” section of the Pew American Values Survey.^42^ Greater values for these items reflect a belief in positive perceptions of government or a more expansive role of government. This construct of government perceptions demonstrated moderate internal consistency across subsamples (Cronbach's α = .55). We constructed our variables for welfare stigma using the “treatment welfare stigma” scale as described by Stuber and colleagues.^34,35^ This construct of welfare stigma demonstrated high internal consistency across subsamples (Cronbach's α = .76). We constructed our variables for discrimination using select items from the Racial Microaggressions Scale.^43^ The construct of discrimination demonstrated high internal consistency across subsamples (Cronbach's α = .81). For each construct, we conducted polychoric principal components analysis using the full-scale responses for each item.^44^ Using the criterion of an eigenvalue greater than 1.0, we retained 2 principal components each for our constructs of government perceptions and welfare stigma and 1 principal component for our construct of discrimination (Supplemental Text). The 2 principal components for government perceptions described a belief in (1) a more expansive role of government and (2) a positive perception of government, respectively. The 2 principal components for welfare stigma described (1) stigma originating from the process of applying or participating in safety-net programs (program stigma) and (2) stigma derived from the perception and anticipation of judgment from others (social stigma) (Supplemental Text, Tables S1–S3). Take-up was measured as (1) self-reported participation in SNAP at the time of interview and (2) receipt of the EITC for tax year 2019, as verified by tax returns.

### Analysis

We conducted multivariable logistic regressions to assess the associations of government perceptions, welfare stigma, and discrimination with participation in SNAP and receipt of the EITC. We adjusted analyses for potential confounders, including respondent characteristics (age, gender, race and ethnicity, education, and marital status), whether the household experienced a reduction in income during the COVID-19 pandemic, household income, number of children in the household, food security status, and participation in WIC. We also conducted a sensitivity analysis among SNAP-eligible respondents reporting any food insecurity. We report the odds ratio (OR) and 95% confidence interval for each association. Analyses were conducted in Stata/SE 17.0 (StataCorp, College Station, TX).

### Ethics approval

This study received ethical approval from the Committees for the Protection of Human Subjects for the State of California and at the University of California Berkeley.

## Results

The final SNAP-eligible subsample included 489 study respondents, consisting of 283 (57.9%) SNAP participants and 206 (42.1%) nonparticipants (Table 1). SNAP participants and nonparticipants were similar in age, gender, educational attainment, reporting a reduction in income during the COVID-19 pandemic, and experiences of food insecurity. Compared with nonparticipants, SNAP participants had lower household income, were less likely to be legally married, and had more children in the household. SNAP participants were less likely to identify as Latinx or Hispanic compared with nonparticipants (50.9% vs 69.9%). Meanwhile, participation in WIC was lower among SNAP participants than nonparticipants (74.2% vs 83.0%). In contrast, receipt of the EITC was higher among SNAP participants than nonparticipants (57.2% vs 51.5%) (Table 1). The distribution of demographic characteristics among SNAP-eligible respondents reporting any food insecurity was similar to that of the primary SNAP-eligible sample (Table S4).

**Table 1.** Descriptive characteristics among SNAP-eligible and EITC-eligible subsamples.

	SNAP-eligible^a^			EITC-eligible^b^		
	Overall (*n* = 489)	Participants (*n* = 283)	Nonparticipants (*n* = 206)	Overall (*n* = 326)	Recipients (*n* = 275)	Nonrecipients (*n* = 51)
Characteristic						
Age, mean (SD), years	32.21 (6.75)	31.81 (6.51)	32.76 (7.05)	32.36 (6.70)	32.86 (6.60)	29.69 (6.69)
Income (10 000’s of USD),^c^ mean (SD)	2.11 (1.53)	1.61 (1.25)	2.78 (1.63)	2.09 (1.26)	2.11 (1.22)	1.98 (1.47)
Female, *n* (%)	461 (94.3%)	267 (94.3%)	194 (94.2%)	307 (94.2%)	258 (93.8%)	49 (96.1%)
Latinx or Hispanic, *n* (%)	288 (58.9%)	144 (50.9%)	144 (69.9%)	180 (55.2%)	140 (50.9%)	40 (78.4%)
Bachelor's degree or greater, *n* (%)	94 (19.2%)	48 (17.0%)	46 (22.3%)	69 (21.2%)	58 (21.1%)	11 (21.6%)
Legally married, *n* (%)	151 (30.9%)	65 (23.0%)	86 (41.7%)	93 (28.5%)	75 (27.3%)	18 (35.3%)
Reduction in income during COVID-19 pandemic, *n* (%)	327 (66.9%)	194 (68.6%)	133 (64.6%)	219 (67.2%)	179 (65.1%)	40 (78.4%)
Number of children in household, *n* (%)						
1-2	305 (62.4%)	166 (58.7%)	139 (67.5%)	195 (59.8%)	163 (59.3%)	32 (62.7%)
3+	184 (37.6%)	117 (41.3%)	67 (32.5%)	131 (40.2%)	112 (40.7%)	19 (37.3%)
Safety-net program participation, *n* (%)						
Participating in SNAP				191 (58.6%)	162 (58.9%)	29 (56.9%)
Participating in WIC	381 (77.9%)	210 (74.2%)	171 (83.0%)	255 (78.2%)	208 (75.6%)	47 (92.2%)
Received EITC in 2019	268 (54.8%)	162 (57.2%)	106 (51.5%)			
Any food insecure, n (%)	230 (47.0%)	134 (47.3%)	96 (46.6%)	145 (44.5%)	118 (42.9%)	27 (52.9%)

Abbreviations: EITC, Earned Income Tax Credit; SNAP, Supplemental Nutrition Assistance Program; WIC, Special Supplemental Nutrition Program for Women, Infants, and Children.

^a^SNAP-eligible at time of interview. Eligibility was estimated using collected data on participant's income and household composition.

^b^EITC-eligible for tax year 2019. Eligibility determined using participant’s filed tax returns from tax year 2019.

^c^Adjusted gross income from 2019 tax returns when available. Household income was self-reported otherwise.

The final EITC-eligible subsample included 326 study respondents, consisting of 275 (84.4%) EITC recipients and 51 (15.6%) nonrecipients, verified from tax returns (Table 1). Compared with nonrecipients, EITC recipients were similar with respect to household income, gender, educational attainment, marital status, number of children in the household, and experiences of food insecurity. The EITC recipients were more likely to be older, less likely to be Latinx or Hispanic (50.9% vs 78.4%), and less likely to report a reduction in income during the COVID-19 pandemic (65.1% vs 78.4%) compared with nonrecipients. When assessing concurrent participation in other safety-net programs, participation in WIC was lower among EITC recipients than nonrecipients (75.6% vs 92.2%) (Table 1).

In multivariable analyses, participating in SNAP at the time of interview was associated with significantly greater odds of experiencing social stigma (OR = 1.24 [1.05, 1.47]), but not with perceptions of government, discrimination, or program stigma (Table 2). Receiving the EITC was also associated with significantly greater odds of experiencing social stigma (OR = 1.39 [1.05, 1.84]) but not with perceptions of government, discrimination, or program stigma (Table 2). In the sensitivity analysis of SNAP-eligible respondents reporting any food insecurity, participating in SNAP at the time of interview was associated with significantly greater odds of experiencing social stigma (OR = 1.31 [1.05, 1.65]), program stigma (OR = 1.19 [1.05, 1.35]), and discrimination (OR = 1.25 [1.07, 1.47]), but not with perceptions of government (Table S5).

**Table 2.** Associations of government perceptions, welfare stigma, and discrimination with safety-net program participation/receipt.^a,b^

	OR (95% CI)	
	SNAP participants vs nonparticipants	EITC recipients vs nonrecipients
Government perceptions		
Principal component 1: More expansive role of government	0.89 (0.78, 1.02)	1.08 (0.86, 1.35)
Principal component 2: Positive perception of government	1.01 (0.86, 1.18)	0.87 (0.67, 1.13)
Welfare stigma		
Principal component 1: Program stigma	1.01 (0.91, 1.11)	1.06 (0.91, 1.24)
Principal component 2: Social stigma	1.24* (1.05, 1.47)	1.39* (1.05, 1.84)
Discrimination		
Principal component 1: Frequent experiences of discrimination	1.14 (1.00, 1.29)	0.96 (0.79, 1.17)

^a^Models adjusted for participant characteristics (ie, age, gender, race and ethnicity, education, marital status), reduction in income during COVID-19 pandemic, household income, number of children in the household, food insecurity, and participation in WIC. ^*^P < .05.

^b^n ranges from 467 to 476 for SNAP participants and from 310 to 317 for EITC participants, depending on model.

Abbreviations: EITC, Earned Income Tax Credit; OR, odds ratio; SNAP, Supplemental Nutrition Assistance Program; WIC, Special Supplemental Nutrition Program for Women, Infants, and Children.

## Discussion

This study examined whether participation in SNAP and receipt of the EITC were associated with perceptions of the US government, 2 types of welfare stigma (program stigma and social stigma), and perceived discrimination among a sample of caregivers of young children from families with low incomes in California. Our key findings were that SNAP participants and EITC recipients were more likely to perceive social stigma but did not report differences in perceptions of government, program stigma, or discrimination. Among food-insecure respondents, we found that participation in SNAP was additionally associated with program stigma and discrimination. These are 2 of the largest US safety-net programs, with low take-up in California relative to national take-up, and different methods of applying for and receiving benefits.

The observed association of SNAP participation with social stigma is consistent with previous literature that found that SNAP participants report anticipating judgment or poor treatment. Contemporary EBT cards (similar in appearance to a debit or credit card) are less visible symbols of government assistance than traditional food stamps. While the universal implementation of EBT has reduced stigma (and has even demonstrated a small positive effect on take-up), it does not completely eliminate stigma associated with the use of SNAP.^30^ SNAP participants report feeling devalued and embarrassed when using their benefits and often feel judged by others, including grocery store cashiers, other shoppers, elected leaders, and the press.^45-48^ This suggests that negative public attitudes about SNAP participants persist and participants themselves are aware of how they are perceived. The social stigma associated with SNAP can be described as a “controllable stigma” (ie, stigma that is perceived to be self-imposed and escapable).^49^ People experiencing a controllable stigma are often blamed for their own position, and public attitudes are generally less sympathetic towards members of these groups.^49^ This appears to be true for SNAP participants in the United States, where people facing financial hardship are often blamed for their own poverty and receive little sympathy from the general public.^50^ Initiatives that target and dispel this framing can be one intervention point for policymakers to help reduce stigma regarding SNAP. Our sensitivity analysis among SNAP-eligible respondents reporting any food insecurity additionally revealed that SNAP participants were more likely to experience program stigma and discrimination than eligible nonparticipants. This may suggest that the perceptions of program stigma and discrimination are amplified with greater need for assistance (ie, any food insecurity), although this finding originates from a nonrepresentative subsample of our data and requires further inquiry.

Our finding that receipt of the EITC is associated with greater social stigma is not consistent with previous literature, which suggests that EITC can actually promote feelings of inclusion.^51^ The EITC offers a lump-sum payment through a system administered by the Internal Revenue Service. Every tax filer in the nation engages with this distribution system, making it impossible to ascertain an individual's receipt of the EITC without reviewing their financial documents. Many recipients are often unaware that they received the benefit,^41^ but among those who are, the EITC is widely viewed as a “deserved reward for hard work.”^30^ This framing promotes social inclusion, self-sufficiency, and positive contributions to society, which is dramatically different than the public attitudes regarding SNAP. Because our survey did not explicitly assess social stigma attributable to the EITC, it is possible that EITC recipients reported experiencing stigma from participation in other safety-net programs or perceiving social stigma more generally.

This study has key strengths, including rich interviews with a sample of California families with low income with detailed information on safety-net program participation and its potential correlates at a time of great economic hardship. This study also has several limitations. First, the original recruitment for the ACCESS study sought to enroll EITC recipients and used convenience sampling methods. The study sample is therefore not representative of the broader SNAP- or EITC-eligible populations in California. We excluded those who reported filing taxes but did not have their tax returns available because we could not verify their EITC receipt. Relatedly, we were unable to assess the net income test for households for SNAP eligibility as this would have required information such as countable resources and appropriate deductions that we did not collect; this may have resulted in misclassification. Second, our study is a correlational analysis due to its cross-sectional design. Our observed associations may reflect reverse causation, in which an increased perception of stigma is responsible for take-up rather than our current interpretation that take-up is contributing to participant stigma. Furthermore, our findings could be subject to unmeasured confounding. We did not investigate interaction among key covariates due to cell sizes. Notably, this study was conducted during the COVID-19 pandemic and in California, which has a high poverty rate and a high percentage of people of color and immigrants. Our findings may not be generalizable to other time periods or states. Future research can remedy these challenges by using a larger, more representative sample of eligible participants and nonparticipants.

Additional avenues of research include examining other reasons for low take-up (eg, insufficient benefits, lack of information, confusion on eligibility) as well as changes in government perceptions, welfare stigma, and discrimination over time (ie, pre-pandemic, during the pandemic, and post-pandemic). Temporary expansions to safety-net programs at the onset of the pandemic resulted in greater levels of assistance to beneficiaries.^18^ These expansions also resulted in increased flexibility in program rules that may have resulted in increased access and reduced program stigma. For example, some states temporarily waived interview requirements and adopted less intensive recertification processes for SNAP in 2020.^52^ Accompanying widespread messaging and greater overall economic need may have also influenced social stigma and perceptions of government. It is unknown whether attitudes will shift, and further research is warranted to measure these changes as temporary pandemic-era expansions to safety-net programs expire.

## Conclusion

Our study finds that participants in two of the largest US safety-net programs experienced greater social stigma than eligible nonparticipants during the early COVID-19 pandemic. Stigma can cause stress, hinder access to resources, and disrupt health-promoting behaviors, which can negatively impact both mental and physical health.^53^ A key recommendation that emerges from our study is to address prevailing social attitudes through improved communications and outreach to reduce social stigma regarding these programs. For example, an Obama-era initiative that encouraged retailers to advertise that they welcomed SNAP reduced stigma and learning costs associated with the program.^30^ An annual EITC awareness day also encourages community organizations, elected officials, schools, employers, and other interested parties to promote awareness of the program.^54^ Low take-up of SNAP and EITC in California leaves many families struggling financially despite their eligibility for benefits. Especially during times of public health and economic crises, safety-net programs that serve critical roles in supporting families can be better deployed if barriers to take-up, such as associated stigma, are reduced.

## Supplementary Material

qxad084_Supplementary_Data
