# Supplementary material for: The association of safety-net program participation with government perceptions, welfare stigma, and discrimination
Source: Health Aff Sch. 2023 Dec 21;2(1):qxad084. doi: 10.1093/haschl/qxad084 (PMC10986270; doi:10.1093/haschl/qxad084)
Supplement: qxad084_Supplementary_Data [file qxad084_Supplementary_Data.zip › snp_access_supplemental_103023.docx]

# SUPPLEMENTAL TEXT

## POLYCHORIC PRINCIPAL COMPONENT ANALYSIS

Similar to principal component analysis, polychoric principal component analysis is an appropriate method to reduce the number of dimensions of our constructs because it does not require each item to be normally distributed and respects the ordinality of the response scale. We retained any principal components with an eigenvalue greater than 1.0 for our analyses (Supplemental Table 2). This criterion yields two principal components each for government perceptions and welfare stigma and one principal component for discrimination.

For principal component 1 of government perceptions, we observed moderate loadings from four items (i.e. it is the responsibility of government to take care of people who can’t take care of themselves; the government should help more needy people even if it means going deeper in debt; the government should guarantee every citizen enough to eat and a place to sleep; and the government needs to do more to make health care affordable and accessible). Principal component 1 of government perceptions described a belief in a more expansive role of government to assist people in need. For principal component 2 of government perceptions, we observed heavy loadings from two items (i.e. when something is run by the government it is usually efficient and not wasteful; and the government is really run for the benefit of the people). Principal component 2 of government perceptions described a positive perception of government, characterizing the efficiency and benevolence of government (Supplemental Table 3). These two principal components for government perceptions explained roughly 60% of the variance in the data (Supplemental Table 2).

For principal component 1 of welfare stigma, we observed moderate loadings from four items (i.e. the application process for welfare is humiliating; many people are treated poorly when they apply for welfare; when applying for welfare, you have to answer unfair questions about your personal life; and when participating in welfare, the rules take away your personal freedom). Principal component 1 of welfare stigma described stigma originating from the process of applying or participating in safety net programs, which we refer to as program stigma. For principal component 2 of welfare stigma, we observed moderate loadings from two items (i.e. many people on welfare do not want other people to know they are on welfare; and there are a lot of people in this country who do not respect a person on welfare). Principal component 2 of welfare stigma described social stigma derived from the perception and anticipation of judgment from others (Supplemental Table 3). These two principal components for welfare stigma explained roughly 68% of the variance in the data (Supplemental Table 2).

For principal component 1 of discrimination, we observed moderate loadings from all six items (Supplemental Table 3). Principal component 1 of discrimination can be interpreted as a unidimensional construct describing perceived discrimination and explained 63% of the variance in the data (Supplemental Table 2).

**Supplemental Table 1. Variable definitions for government perceptions, welfare stigma, and discrimination constructs.**

|  |  |  | **Cronbach's Alpha^a^** | |
| --- | --- | --- | --- | --- |
| **Construct Items** | | **Variable Structure** | **Among SNAP-Eligible** | **Among EITC-Eligible** |
| Government perceptions | |  |  |  |
|  | 1) When something is run by the government it is usually efficient and not wasteful.^b^ | Responses ranged from strongly disagree (1) to strongly agree (5) on a 5-point Likert scale. Principal components calculated using polychoric principal component analysis. | 0.5548 | 0.5527 |
|  | 2) The government is really run for the benefit of the people. |  |  |  |
|  | 3) It is the responsibility of the government to take care of people who can't take care of themselves. |  |  |  |
|  | 4) The government should help more needy people even if it means going deeper in debt. |  |  |  |
|  | 5) The government should guarantee every citizen enough to eat and a place to sleep. |  |  |  |
|  | 6) The government needs to do more to make health care affordable and accessible. |  |  |  |
|  |  |  |  |  |
| Welfare stigma | |  |  |  |
|  | 1) The application process for welfare is humiliating. | Responses ranged from strongly disagree (1) to strongly agree (5) on a 5-point Likert scale. Principal components calculated using polychoric principal component analysis. | 0.7630 | 0.7707 |
|  | 2) Many people are treated poorly when they apply for welfare. |  |  |  |
|  | 3) When applying for welfare, you have to answer unfair questions about your personal life. |  |  |  |
|  | 4) When participating in welfare, the rules take away your personal freedom. |  |  |  |
|  | 5) Many people on welfare do not want other people to know they are on welfare. |  |  |  |
|  | 6) There are a lot of people in this country who do not respect a person on welfare. |  |  |  |
|  |  |  |  |  |
| Discrimination | |  |  |  |
|  | 1) Other people treat me like a criminal and/or act like they are scared of me. | Responses ranged from never (1) to often/frequently (4). Principal components calculated using polychoric principal component analysis. | 0.8112 | 0.8258 |
|  | 2) Others only focus on the negative aspects of my racial background. |  |  |  |
|  | 3) Others hint that I should work hard to prove that I am not like other people of my race. |  |  |  |
|  | 4) Other people act as if all people of my race are alike. |  |  |  |
|  | 5) Others assume that people of my background would succeed in life if they simply worked harder. |  |  |  |
|  | 6) I receive poorer treatment in restaurants and stores because of my race. |  |  |  |

Abbreviations: SNAP (Supplemental Nutrition Assistance Program), EITC (Earned Income Tax Credit)

^a^ Cronbach's alpha measures internal consistency and can be used as a measure of scale reliability.
^b^ Survey question asked "When something is run by the government it is usually inefficient and wasteful" and was reversed in analysis to mirror the coding and direction of other component variables used to construct the government perceptions variable.

**Supplemental Table 2. Polychoric principal component analysis: eigenvalues and proportion of variance explained.**

| **Principal Components** | | **Eigenvalue** | **Proportion of  variance explained** | **Cumulative  variance explained** |
| --- | --- | --- | --- | --- |
| Government perceptions | |  |  |  |
|  | Component 1 | 2.3664 | 0.3944 | 0.3944 |
|  | Component 2 | 1.2823 | 0.2137 | 0.6081 |
|  | Component 3 | 0.8381 | 0.1397 | 0.7478 |
|  | Component 4 | 0.6108 | 0.1018 | 0.8496 |
|  | Component 5 | 0.4746 | 0.0791 | 0.9287 |
|  | Component 6 | 0.4278 | 0.0713 | 1.0000 |
|  |  |  |  |  |
| Welfare stigma | |  |  |  |
|  | Component 1 | 2.9993 | 0.4999 | 0.4999 |
|  | Component 2 | 1.1035 | 0.1839 | 0.6838 |
|  | Component 3 | 0.5553 | 0.0925 | 0.7764 |
|  | Component 4 | 0.5388 | 0.0898 | 0.8661 |
|  | Component 5 | 0.4571 | 0.0762 | 0.9423 |
|  | Component 6 | 0.3460 | 0.0577 | 1.0000 |
|  |  |  |  |  |
| Discrimination | |  |  |  |
|  | Component 1 | 3.7792 | 0.6299 | 0.6299 |
|  | Component 2 | 0.6976 | 0.1163 | 0.7461 |
|  | Component 3 | 0.4808 | 0.0801 | 0.8263 |
|  | Component 4 | 0.4313 | 0.0719 | 0.8982 |
|  | Component 5 | 0.4029 | 0.0671 | 0.9653 |
|  | Component 6 | 0.2082 | 0.0347 | 1.0000 |

**Supplemental Table 3. Variable loadings in polychoric principal component analysis for government perceptions, welfare stigma, and discrimination.**

|  |  | **Variable Loading** | |
| --- | --- | --- | --- |
| **Construct Items** | | **PC1** | **PC2** |
| Government perceptions | |  |  |
|  | 1) When something is run by the government it is usually efficient and not wasteful.^a^ | -0.1454 | 0.6256 |
|  | 2) The government is really run for the benefit of the people. | -0.0694 | 0.7290 |
|  | 3) It is the responsibility of the government to take care of people who can't take care of themselves. | 0.4794 | 0.2387 |
|  | 4) The government should help more needy people even if it means going deeper in debt. | 0.5179 | 0.0772 |
|  | 5) The government should guarantee every citizen enough to eat and a place to sleep. | 0.5284 | 0.0613 |
|  | 6) The government needs to do more to make health care affordable and accessible. | 0.4436 | -0.1021 |
|  |  |  |  |
| Welfare stigma | |  |  |
|  | 1) The application process for welfare is humiliating. | 0.4177 | -0.2535 |
|  | 2) Many people are treated poorly when they apply for welfare. | 0.4534 | -0.1142 |
|  | 3) When applying for welfare, you have to answer unfair questions about your personal life. | 0.4281 | -0.3323 |
|  | 4) When participating in welfare, the rules take away your personal freedom. | 0.4474 | -0.2649 |
|  | 5) Many people on welfare do not want other people to know they are on welfare. | 0.3317 | 0.6190 |
|  | 6) There are a lot of people in this country who do not respect a person on welfare. | 0.3557 | 0.5991 |
|  |  |  |  |
| Discrimination | |  |  |
|  | 1) Other people treat me like a criminal and/or act like they are scared of me. | 0.3868 | - |
|  | 2) Others only focus on the negative aspects of my racial background. | 0.4521 | - |
|  | 3) Others hint that I should work hard to prove that I am not like other people of my race. | 0.4020 | - |
|  | 4) Other people act as if all people of my race are alike. | 0.4195 | - |
|  | 5) Others assume that people of my background would succeed in life if they simply worked harder. | 0.3732 | - |
|  | 6) I receive poorer treatment in restaurants and stores because of my race. | 0.4113 | - |

^a^ Survey question asked "When something is run by the government it is usually inefficient and wasteful" and was reversed in analysis to mirror the coding and direction of other component variables used to construct the government perceptions variable.

**Supplemental Table 4. Descriptive characteristics among SNAP-eligible and food insecure participants.**

|  |  | **SNAP-Eligible^a^** | | |
| --- | --- | --- | --- | --- |
|  |  | **Overall** | **Participants** | **Non-participants** |
| **Characteristic** | | **N = 489** | **N = 283** | **N = 206** |
| Age (years), mean (SD) | | 31.91 (6.67) | 31.34 (6.42) | 32.72 (6.95) |
| Income (10,000s of USD)^b^, mean (SD) | | 2.03 (1.56) | 1.55 (1.26) | 2.70 (1.69) |
| Female, n (%) | | 218 (94.8%) | 127 (94.8%) | 91 (94.8%) |
| Latinx or Hispanic, n (%) | | 144 (62.6%) | 75 (56.0%) | 69 (71.9%) |
| Bachelor's degree or greater, n (%) | | 33 (14.3%) | 20 (14.9%) | 13 (13.5%) |
| Legally married, n (%) | | 66 (28.7%) | 28 (20.9%) | 38 (39.6%) |
| Decrease in income during COVID-19 pandemic, n (%) | | 172 (74.8%) | 97 (72.4%) | 75 (78.1%) |
| Number of children in household, n (%) | |  |  |  |
|  | 1-2 | 136 (59.1%) | 71 (53.0%) | 65 (67.7%) |
|  | 3+ | 94 (40.9%) | 63 (47.0%) | 31 (32.3%) |
| **Safety Net Program Participation & Food Security Status** | |  |  |  |
| Participating in SNAP, n (%) | |  |  |  |
| Participating in WIC, n (%) | | 182 (79.1%) | 94 (70.1%) | 88 (91.7%) |
| Received EITC in 2019, n (%) | | 115 (50.0%) | 70 (52.2%) | 45 (46.9%) |

Abbreviations: SNAP (Supplemental Nutrition Assistance Program), EITC (Earned Income Tax Credit), WIC (Special Supplemental Nutrition Program for Women, Infants, and Children)

^a^ SNAP-eligible at time of interview. Eligibility was estimated using collected data on participant's income and household composition.
^b^ Adjusted gross income from 2019 tax returns when available. Household income was self-reported otherwise.

**Supplemental Table 5. Associations of government perceptions, welfare stigma, and discrimination with safety net program participation among SNAP eligible and food insecure participants.^a^**

|  | **SNAP Participants  vs. Non-Participants** |
| --- | --- |
|  | **N = 228** |
| **Government Perceptions** | **OR (95% CI)** |
| Principal component 1: More expansive role of government | 1.07 (0.90, 1.28) |
| Principal component 2: Positive perception of government | 0.96 (0.79, 1.17) |
|  |  |
|  | **SNAP Participants  vs. Non-Participants** |
|  | **N = 227** |
| **Welfare Stigma** | **OR (95% CI)** |
| Principal component 1: Stigma regarding the process of applying/participating | 1.19* (1.05, 1.35) |
| Principal component 2: Social stigma | 1.31* (1.05, 1.65) |
|  |  |
|  | **SNAP Participants  vs. Non-Participants** |
|  | **N = 224** |
| **Discrimination** | **OR (95% CI)** |
| Principal component 1: Frequent experiences of discrimination | 1.25* (1.07, 1.47) |
|  |  |

* p<0.05

Abbreviations: SNAP (Supplemental Nutrition Assistance Program)

^a^ Models adjusted for participant characteristics (i.e. age, gender, race and ethnicity, education, marital status), decrease in income during COVID-19 pandemic, household income, number of children in the household, and participation in WIC.
